# Comprehensive Approaches to Combatting *Acinetobacter baumannii* Biofilms: From Biofilm Structure to Phage-Based Therapies

**DOI:** 10.3390/antibiotics13111064

**Published:** 2024-11-08

**Authors:** Ilona Grygiel, Olaf Bajrak, Michał Wójcicki, Klaudia Krusiec, Ewa Jończyk-Matysiak, Andrzej Górski, Joanna Majewska, Sławomir Letkiewicz

**Affiliations:** 1 Bacteriophage Laboratory, Hirszfeld Institute of Immunology and Experimental Therapy, Polish Academy of Sciences, 53-114 Wroclaw, Poland; ilona.grygiel@hirszfeld.pl (I.G.); olaf.bajrak@hirszfeld.pl (O.B.); michal.wojcicki@hirszfeld.pl (M.W.); k.krusiec.97@gmail.com (K.K.); andrzej.gorski@hirszfeld.pl (A.G.); 2 Phage Therapy Unit, Hirszfeld Institute of Immunology and Experimental Therapy, Polish Academy of Sciences, 53-114 Wroclaw, Poland; letkiewicz1@o2.pl; 3 Professor Emeritus, Department of Immunology, The Medical University of Warsaw, 02-006 Warsaw, Poland; 4 Department of Pathogen Biology and Immunology, University of Wrocław, 51-148 Wrocław, Poland; joanna.majewska@uwr.edu.pl; 5 Collegium Medicum, Jan Długosz University, 42-200 Częstochowa, Poland

**Keywords:** *Acinetobacter baumannii*, antibiotic resistance, biofilm formation and eradication, bacteriophage (phage), phage therapy, depolymerases

## Abstract

*Acinetobacter baumannii*—a multidrug-resistant (MDR) pathogen that causes, for example, skin and soft tissue wounds; urinary tract infections; pneumonia; bacteremia; and endocarditis, particularly due to its ability to form robust biofilms—poses a significant challenge in clinical settings. This structure protects the bacteria from immune responses and antibiotic treatments, making infections difficult to eradicate. Given the rise in antibiotic resistance, alternative therapeutic approaches are urgently needed. Bacteriophage-based strategies have emerged as a promising solution for combating *A. baumannii* biofilms. Phages, which are viruses that specifically infect bacteria, offer a targeted and effective means of disrupting biofilm and lysing bacterial cells. This review explores the current advancements in bacteriophage therapy, focusing on its potential for treating *A. baumannii* biofilm-related infections. We described the mechanisms by which phages interact with biofilms, the challenges in phage therapy implementation, and the strategies being developed to enhance its efficacy (phage cocktails, engineered phages, combination therapies with antibiotics). Understanding the role of bacteriophages in both biofilm disruption and in inhibition of its forming could pave the way for innovative treatments in combating MDR *A. baumannii* infections as well as the prevention of their development.

## 1. Introduction

*Acinetobacter baumannii* is a Gram-negative non-motile, rod-shaped (coccobacillus), opportunistic human pathogen from the *Moraxellaceae* family. *A. baumannii* is widely spread through the environment, food, and medical devices [1]. Furthermore, it can also be isolated from humans and animals (e.g., cats, dogs, horses, and insect vectors) [2,3,4]. Pathogens colonizing the surface of medical devices, e.g., ventilators and catheters, may cause serious infections, especially in immunocompromised individuals [1,5,6]. *A. baumannii* is known to reside in the human microbiota (e.g., the digestive tract), especially after hospital visits in patients. Moreover, it may cause pneumonia, urinary tract infections (UTIs), and other nosocomial infections that are frequently acquired in a hospital setting, especially in intensive care units (ICUs) [4,7]. *A. baumannii* may also be responsible for meningitis [8], skin conditions (wound infections, exudates, and abscesses), local infections (osteomyelitis and endophthalmitis), or bloodstream infections (bacteremia, endocarditis) [4,7]. After a prolonged stay in a hospital, *A. baumannii* may colonize the patient’s body, becoming a harmless component of the microbiota. In a study based on the colonization of human skin and mucous membranes by *Acinetobacter* spp., conducted on 30 hospitalized subjects and 17 healthy individuals, it was observed that *Acinetobacter* strains were present in the composition of the microbiota of as many as 43% of people who had not been treated in hospitals. The microbiota of hospitalized patients was dominated by strains of the *Acinetobacter* genus (about 75% of strains), including *Acinetobacter lwoffii* (47%) and *Acinetobacter johnsonii* (21%), while *A. baumannii* accounted only for 1.5%. Nevertheless, the species most frequently isolated from clinical specimens, such as blood, urine, cerebrospinal fluid, wound, and throat specimens, among others, is *A. baumannii* [9].

## 2. *Acinetobacter baumannii* as a Priority Bacterial Pathogen

*Acinetobacter baumannii* belongs to the ESKAPE group (*Enterococcus faecium*, *Staphylococcus aureus*, *Klebsiella pneumoniae*, *Acinetobacter baumannii*, *Pseudomonas aeruginosa*, and *Enterobacter* spp.). These highly virulent bacteria effectively ‘escape’ commonly used antimicrobial treatments due to the dramatically increasing number of more and more sophisticated mechanisms of resistance to different classes of antibiotics [1,6,7,10]. Carbapenem-resistant *A. baumannii* has been recognized by the World Health Organization (WHO) as one of the 12 top-priority antibiotic-resistant pathogens, for which new and effective antibiotics or alternative therapeutic options are urgently needed. It was classified within the first, most critical priority group [11]. Moreover, bacteria from the *Acinetobacter* genus have also been reported as the 14th most common pathogen within care facilities and hospitals [4], and MDR *A. baumannii* strains are now considered endemic and epidemic in hospitals worldwide [12]. This indicates that this bacterial pathogen poses a major threat, especially given its resistance to factors such as UV light, drying, and disinfectants [13,14,15].

Currently, resistance to all known and described antibiotics, such as β-lactams (including carbapenems), peptide antibiotics, tetracyclines, aminoglycosides, and quinolones, is observed among *A. baumannii* strains [16]. Bacterial strains contain markers indicating resistance to carbapenems as well as to other classes of antibiotics. Carbapenem-resistant strains of *A. baumannii* are prevalent in hospitals in Asia and North and South America. The resistance rate [R%] (defined as the number of carbapenem-resistant *A. baumannii* (CRAB) strains divided by the number of all samples from which *A. baumannii* strains were isolated) in these areas is almost 90%, while in Scandinavia and east-central Europe, it is much lower, at less than 10% [17]. Considering the prevalence of CRAB strains in 2021, it is essential to mention the Multi-Profile Hospital in Wrocław (Poland), where it was estimated that infections caused by *A. baumannii* reached their highest level in the last 6 years [18]. Assuming that approximately 70% of these strains were MDR [19], this pathogen is an enormous threat amongst MDR pathogens in Poland. The sharp increase in resistance among *A. baumannii* strains was observed after the COVID-19 pandemic because antibiotic therapy used at that time was frequently prescribed unjustifiably [20,21].

Besides the ability to acquire new and much more sophisticated mechanisms of antimicrobial resistance, another reason for *A. baumannii* being so dangerous and difficult to eradicate is its ability to form a biofilm. Depending on the location of the strains and their origin, this ability differs—clinical strains (which exist almost exclusively in hospital environments) generally have a higher capability to produce biofilms than environmental ones [1,7]. Moreover, *A. baumannii* is the most common microbe that can form a biofilm found in drinking-water pipe systems. Major epidemic clones produce stronger biofilms than minor and sporadic clones, which is related to the expression of virulence factors. In the planktonic state, minor clones express more gene determinants associated with the virulence mechanisms than major epidemic clones [7].

The first line of antibacterial immune defense against invading *A. baumannii* involves neutrophils, macrophages, the complement system, and antimicrobial peptides (AMPs) [6]. Neutrophils generally destroy bacteria using different mechanisms, such as phagocytosis, degranulation, and NETosis, which involves neutrophil cell death and, consequently, the release of neutrophil extracellular traps (NETs) consisting of web-like chromatin structures decorated with antibacterial factors that may bind pathogens. Interestingly, it was demonstrated that, unlike many other Gram-negative bacteria, *A. baumannii* can impede NETosis. *A. baumannii* clearance by phagocytosis involves the generation of reactive oxygen species (ROS) by the nicotinamide adenine dinucleotide phosphate (NADPH) oxidase within the neutrophils [22]. Macrophages exhibit a minor role during *A. baumannii* infection. The last element of the immune system which acts against *A. baumannii* is the complement system. This system consists of more than 30 plasma proteins that contribute as a cascade, triggering bacterial cell lysis or opsonization and phagocytosis. Of the three complement activation pathways (classical, lectin, and alternative), the alternative pathway appears to play the dominant—if not exclusive—role in the neutralization of *A. baumannii* [6]. Sanchez-Larrayoz and co-workers (2017) found 50 genes that are required for the survival of *A. baumannii* in human serum. Many of these genes encode Mla proteins that prevent the accumulation of phospholipids in the bacterial outer membrane (OM). The role of the Mla pathway is to maintain the asymmetry of the bacterial OM by transporting accumulated phospholipids to the inner membrane (IM). The same scientific team has confirmed the effective action of the Mla pathway in protecting *A. baumannii* from the host immune system [23]. Strains that are deficient in Mla proteins and thus accumulate phospholipids in the OM are recognized by the human complement system [23,24]. A consequence of complement activation, regardless of the activation pathway, is the formation of a membrane attack complex (MAC) that attacks the membrane, which can lead to direct destruction of bacterial cells.

Surface glycoconjugates are major factors involved in evading the innate immune response by *A. baumannii*, yet other mechanisms are also at play. An exopolysaccharide capsule (formed by a long polysaccharide chain containing repeated K units of carbohydrates) protects *A. baumannii* against the host immune response. This phenomenon may be attributed to the hydrophilicity of capsular polysaccharides (CPSs) and the negative charge of CPS monosaccharides interacting with the negatively charged surfaces of neutrophils and macrophages. The presence of this structure is linked to bacterial virulence and persistence in the host, leading to more severe infections caused by capsule-producing strains and resulting in higher mortality rates [6]. It was demonstrated that phage-resistant *A. baumannii* strains may have loss-of-function mutations in genes of the locus K, which is involved in regulating the production, modification, and export of capsular polysaccharides. Interestingly, it is postulated that the loss of capsules in *A. baumannii* isolates may be associated with a reduced ability to form biofilms and resensitization to antibiotics [25]. It was demonstrated that capsules mediate both cationic antimicrobial peptide resistance and serum resistance [1]. Altamirano and co-workers (2021) also observed that the loss of locus K (deletions in the *gtr29* gene, which encodes glycosyltransferase, and in the *gpi* gene encoding the enzyme glucose-6-phosphate isomerase were studied) resulted in decreased fitness in a mouse model of bacteremia [25]. Moreover, outer membrane/extracellular polymeric substances (OM/EPSs) and OM protein are other barriers protecting *A. baumannii* against the host response. Lipopolysaccharide (LPS), the main component of the outer membrane, contains three domains: lipid A, serving as a membrane anchor; oligosaccharide core; and long-chain O-antigenic polysaccharide. Another component of the membrane is lipooligosaccharide (LOS), whose deficiency contributes to changes in interaction of *A. baumannii* with the host’s innate immune system [6]. LPS also contributes to resistance to cationic AMPs and activates the host inflammatory response [1]. Interestingly, the complete lack of LOS or the addition of galactosamine to LOS can lead to colistin resistance. Such strains are characterized by decreased virulence and increased susceptibility to human cathelicidin, a 37-residue, amphipathic helical peptide (LL-37), and lysozyme [6].

## 3. Mechanisms of Antibiotic Resistance in *A. baumannii*

*A. baumannii* is a significant concern due to its multiple mechanisms of resistance to antibiotics, disinfectants, and desiccation techniques [16,26]. It shows resistance to all first-line antibiotics used in treating infections caused by this threatening pathogen, including β-lactams, aminoglycosides, and quinolones. This microbe possesses 52 resistance genes, 45 of which are found in the AbaR1 genomic antibiotic resistance island, which is an 86 kb region with 88 open reading frames (ORFs) [27]. Interestingly, DNA sequencing reveals that 82 of these ORFs likely originated from other bacterial species, such as *Salmonella* sp., *E. coli*, and *Pseudomonas* sp., mostly through horizontal gene transfer (HGT). Nevertheless, first-line antibiotics are still often used in clinical practice against *A. baumannii*, especially when the isolated strains do not demonstrate extensive antibiotic resistance [28]. Some recent case studies also report successful use of antibiotics in the treatment of pandrug-resistant (PDR) [29] or extensively drug-resistant (XDR) [30] *A. baumannii* infections. However, in these particular cases, the proposed treatment regimen involved either a combination of several antibiotics (including colistin), applied both systemically and locally [29], or a novel catechol siderophore cephalosporin antibiotic cefiderocol [30], which was, at that time, pursued for compassionate use and only later approved for medical use in the United States and Europe.

The largest known group of antibiotics are β-lactams, which include penicillins and carbapenems as well as cephalosporins and monobactams [31]. They are composed of a β-lactam ring, which is characterized by the presence of an amide bond and a carbonyl group. Their main function is to inhibit cell wall biosynthesis in bacteria. *A. baumannii* strains very quickly adapt to the presence of β-lactams in the environment, e.g., by producing enzymes that destroy the β-lactam ring. This is how they gained resistance to all β-lactam antibiotics. In addition, all *A. baumannii* strains encode genes responsible for AmpC cephalosporinase production localized in chromosomal DNA. A DNA fragment found in *A. baumannii* known as the insertion sequence (ISAba) occurs upstream of the gene encoding the cephalosporinase [16]. It regulates its expression contributing to the production of cephalosporinases and is associated with increased bacterial resistance to these antibiotics [32]. Fournier and co-workers (2006) described a region corresponding to 86 kb that contained a cluster of 45 resistance genes in the *A. baumannii* strain AYE [27]. This region was named an “island” of resistance based on its size, high content of GC pairs compared to other genome areas (52.8%), and the presence of transposases, integrases, and insertion genes [33]. It was also discovered that the vast majority of known antibiotic resistance genes are located in this area [27]. Bacteria rapidly acquire resistance to antibiotics using a diverse range of mechanisms, including the production of β-lactamases that cause β-lactam antibiotics to break down [34]. Therefore, inhibitors of these enzymes are also administered along with some antibiotics. These include clavulanic acid, sulbactam and tazobactam.

Other antibiotic-inhibiting enzymes that are also associated with *A. baumannii* strains include metallo-β-lactamases (MBLs). The presence of zinc as a cofactor enables MBLs to function properly [35]. MBL-producing bacteria are resistant to an even larger group of β-lactam antibiotics (including carbapenems) and show resistance to available β-lactamase inhibitors. In addition, MBLs are encoded within plasmids and can be more easily transferred to other bacteria. The mechanism of action of these enzymes involves cleaving the amide bond of the β-lactam ring of the antibiotic [36].

Some enzymes hydrolyze oxacillin, an antibiotic belonging to the penicillin group. This mechanism provides the pathogen with resistance to penicillins [37]. Although *A. baumannii* is known to express this machinery naturally, it was reported that it might acquire genes encoding these proteins from other microorganisms. The first enzyme that was discovered from the oxacillinases group, OXA-23, was found in *A. baumannii* strains. It was reported to have hydrolytic activity in regard to carbapenems [38].

Another mechanism of antibiotic resistance observed in *A. baumannii* is the modification of the binding sites affected by the antibiotic compound. Outer membrane proteins are responsible for binding different antibiotics. Resistance to carbapenems, e.g., imipenem, is associated with the loss of the CarO protein located on the surface of the bacterial OM through the disruption of the *carO* gene by insertion elements [39]. It has been shown that the loss of this protein, along with other surface proteins, such as penicillin-binding proteins (PBPs), may, together with bacterial enzymes, create a common and complementary defense mechanism against antibiotics [39,40]. PBPs are membrane-associated proteins that play a key role in the biosynthesis of peptidoglycan (PG), the primary component of bacterial cell walls [41]. This group of enzymes is essential for the final stages of bacterial cell wall formation, assembling N-acetylglucosamine and N-acetylmuramic acid [42] while also catalyzing the formation of cross-links between individual PG chains. In *A. baumannii*, seven genes have been recognized as being responsible for PBP expression [43]. β-lactam antibiotics bind to PBP, causing inhibition of the synthesis of cell wall components. Conducted studies using an electrophoretic analysis of *A. baumannii* strains, cultured in the presence of ampicillin, indicated the absence of genes responsible for the expression of the PBP2 protein [40]. This suggests that these strains were resistant to this antibiotic.

The resistance of *A. baumannii* to quinolones is caused by mutations in the *parC* and *gyrA* genes encoding type II DNA topoisomerases. A single *gyrA* mutation that induces an amino acid change from serine to leucine at position 83 reduces *A. baumannii* susceptibility to fluoroquinolones. Resistance to these antibiotics also occurs in strains with *parC* mutations—a change of serine to isoleucine at position 80 and glutamic acid to valine at position 84 in the expressed proteins [44].

Colistin acts as a membrane destabilizer in Gram-negative bacteria, ultimately leading to bacterial cell death through targeting and binding the lipid A anchor of LPS or LOS and the outer membrane phospholipids [4,10]. It has been shown that lipid A modification can lead to resistance to colistin in *A. baumannii* isolates [4]. Because of the possibility of causing side effects, such as nephrotoxicity and neurotoxicity, it is used as a last-call antibiotic [10], e.g., for the fight with PDR isolates or for treatment of severe infections, such as meningitis [4].

Furthermore, *A. baumannii* strains have many mechanisms to help pump out antibiotics and/or disinfectants from the cell—the efflux system. Five subfamilies have been characterized so far which play crucial roles in antibiotic resistance: (1) the ATP-binding cassette transporter (ABC transporters), (2) small multidrug resistance (SMR), (3) multi-antimicrobial extrusion protein (or multidrug and toxic compound extrusion, MATE), (4) major facilitator superfamily (MFS), (5) and the resistance-nodulation-division family (RND) [7]. It is responsible for the resistance to a few classes of antibiotics (aminoglycosides—AdeABC; MATE [45,46], quinolones—RND; AdeABC; MFS [46,47,48], tetracyclines—AdeABC [49,50]) and even a disinfectant (chlorhexidine—AceI efflux protein) [13].

## 4. Biofilm—Structure and Development

Current antibiotic treatment options for *A. baumannii* infections were recently discussed in a review by Dolma and co-workers [51]. Nevertheless, these therapeutic approaches are often limited, given the pathogen’s tendency to develop or acquire resistance to a wide range of antibiotics, including β-lactams, aminoglycosides, tetracyclines, macrolides, fluoroquinolones, as well as chloramphenicol, trimethoprim, and colistin [7]. Another limitation of the efficacy of standard antibiotic therapy comes from the prevalence of the genes whose expression enables the biofilm formation. Interestingly, according to a study by Song and co-workers (2015), imipenem, colistin, and rifampicin were not successful at reducing a *A. baumannii* biofilm produced by the VIM-2 strain (resistant to carbapenems) in vitro when used individually, yet a significant reduction in biofilm formation was achieved when the antibiotics were used in combination, suggesting that combination therapy may be a potent approach in treating biofilm-related *A. baumannii* infections [52].

Biofilm is a microbial community that is distinct from planktonic bacteria and adheres to both biotic and abiotic surfaces, such as glass, stainless steel, and plastics, so it contributes to medical-device-associated infections. The forming of biofilms relies on the transformation of bacteria from an exponential to a stationary phase of growth [53]. Biofilms may also be formed under static conditions or within flow cell systems, such as bioreactors where nutrients are supplemented [4]. Additionally, the structure can be formed by binding to host tissues [1,54,55]. Microcolonies act as basic structural units of biofilms, usually comprising roughly 10–25% cells and 75–90% exopolysaccharide (EPS). The *epsA* gene encodes EPS and forms a protective barrier for biofilm from harsh environmental conditions [1,51,55,56]. EPS includes polysaccharides, proteins, nucleic acids, lipids, water, and mineral ions, which protect microorganisms from the external environment, including the host immune system and antimicrobial agents (antibiotics, disinfectants). Polysaccharides are key components linked to a polypeptide chain that consists of negatively charged amino acid side chains. These negatively charged side chains attract positively charged amino acid side chains, creating an environment that hinders the penetration of hydrophilic antibiotics into the bacterial cell [51]. Unfortunately, *A. baumannii* often appears in this form on various medical equipment and devices, and the presence of a polysaccharide envelope makes it difficult to remove. A biofilm of *A. baumannii* is formed on catheters, endotracheal tubes, prostheses, medical instruments, and even on glass surfaces [57,58]. When discussing the antimicrobial resistance mechanisms of ESKAPE pathogens, Santajit and Indrawattana (2016) suggested that biofilm formation could be considered the main cause of antibiotic resistance, proving more relevant to the problem than the classical resistance mechanisms. Indeed, the biofilm matrix not only poses a mechanical barrier in regard to the penetration of antimicrobials but also provides specific environmental conditions, such as low oxygen levels, high CO_2_, low pH, and low water availability, which can altogether hinder the activity of antibiotics. In addition, with low nutrient content determining biofilm formation, antibiotic tolerance can also increase [35].

**Figure 1:**
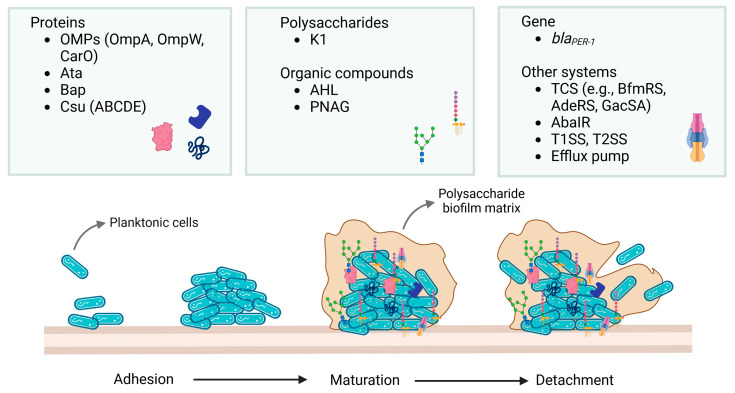
Factors responsible for biofilm formation by *Acinetobacter baumannii* (Biorender, agreement number: HE27IRGOLS).

Available experimental data indicate that the biofilm production rate is much higher for the *A. baumannii* species than for the other, less clinically relevant *Acinetobacter* spp. [59]. The National Institutes of Health (NIH) and the Center for Disease Control and Prevention (CDC) estimated that about 80% of human diseases caused by bacteria are due to biofilm formation [60]. Multiple factors, including physicochemical and microbial determinants such as the aggregation of substances, the adhesion of collagen, the expression of pili, capsular polysaccharides, and resistance determinants, are involved in the production and maintenance of a biofilm. In the process of biofilm formation, three phases are distinguished: adhesion, maturation, and detachment (Figure 1).

During the first phase, planktonic cells are reversibly attached to the surface through weak interactions, and, after the attachment, planktonic cells make bonds. These are more specific molecular interactions between bacterial surface structures and host molecules working as receptors [12]. In addition, the downregulation of motility genes and the up-regulation of adhesins are observed [4]. In the second step, during the biofilm maturation, bacteria produce large amounts of EPS, which form the biofilm’s mass [12]. Furthermore, the bacterial community starts to accumulate as a microcolony, which is an irreversible attachment. Then, cell–cell adhesion occurs with the production of extracellular matrix (ECM) components. In the third step, during the detachment phase, single bacterial cells or cell clusters separate from the biofilm and are released into the surrounding environment and colonize neighboring sites. Some researchers distinguish two additional stages of biofilm development—the fourth being called maturation II or full maturation and the final fifth stage being called dispersion. This fifth stage reinitiates the cycle as signaling promotes the dispersion of the biofilm and cells again enter the planktonic state and disseminate to another place [4]. Administration of antibiotics in concentrations below the minimum inhibitory concentration (MIC) probably increases the formation of biofilms also created by *A. baumannii* strains [12]. Speed and efficiency of attachment for biofilms are affected by surface composition. Bacterial cell surface is negatively charged and therefore more likely to attach to positively charged surfaces. Adhesins can lead to binding to biotic surfaces which may have similar charges [4]. Generally, in the biofilm, factors such as microbial aggregations, overexpression of EPS, alterations in microbial phenotypic and genotypic features (due to stress responses), and physiological heterogeneity (caused by physicochemical gradients and persisters) cause impaired drug diffusion and amplify drug resistance [1,51]. Each bacterium has a unique regulation of biofilms, but there are some universal triggers to initiate the process [4]. During biofilm formation, bacteria can draw in other species, resulting in mixed biofilms [61,62]. In this type of biofilm, antimicrobial resistance can increase by up to 1000 times [63]. Rapid HGT distinguishes biofilms from planktonic cells [64]. Inside biofilms, bacterial evolution and the development of drug-resistant bacteria can be achieved through the transfer of mobile genetic elements that encode antibiotic resistance genes, such as plasmids. MICs of antibiotics added to a forming biofilm drive HGT within it. In this way, biofilms accelerate the formation of antibiotic-resistant bacteria [65]. Penesyan and co-workers (2019) proved that three-day exposure of *A. baumannii* to subinhibitory concentrations of antibiotics increases biofilm formation and the acquisition of antibiotic resistance. The consequence is a change on both a phenotypic and genomic level [66]. In this study, the impact of ciprofloxacin and tetracycline on *A. baumannii* biofilm was examined, predominantly because the investigated strain exhibited a relatively low level of resistance to these antibiotics but also because they represent two different, chemically diverse classes of antimicrobials with a fundamentally different mechanism of action. While ciprofloxacin inhibits DNA gyrase and topoisomerase, ultimately inhibiting cell division [67], tetracycline binds to the 30S ribosomal subunit, inhibiting protein synthesis [66,68]. Importantly, both antibiotics also penetrate bacterial biofilms, as demonstrated by this research as well as other studies, e.g., conducted by Stone and co-workers [69] and Shigeta and co-workers [70]. The results showed that bacterial cells dispersing from antibiotic-exposed biofilms had higher MIC values. This indicates that their resistance increased compared to bacteria whose biofilms were not cultured with antibiotics [66].

### 4.1. Factors Responsible for Biofilm Formation by Acinetobacter baumannii Strains

*A. baumannii* uses different factors or mechanisms to elude the innate immune response [6]. Some of them are involved in the biofilm formation and development in *A. baumannii* strains (Table 1).

The outer membrane protein A (OmpA), other proteins (i.e., biofilm-associated protein (Bap) and the *bap* gene, Bla (PER-1) enzyme, *Acinetobacter* trimeric autotransporter (Ata)), extracellular polysaccharides (LPS, poly-β-1-6-N-acetylglucosamine (PNAG)), K1 capsule, a siderophore-mediated iron-acquisition system, phospholipases (i.e., phospholipase D, phospholipase C), Csu (A/BABCDE and their genes), two-component regulatory system (BfmRS, AdeRS, GacSA, and their genes), *epsA*, six genes of pilus, gene sequences ST 25, ST 78 are indicated as virulence factors that influence the formation of biofilms by *A. baumannii* strains [1,5,51,72]. Polysaccharides participate in biofilm adhesion, providing protection from the host and maintaining its structural integrity [4].

Colquhound and Rather (2020) distinguished 132 biofilm-related genes in *A. baumannii* strains whose knockdowns caused anomalies within outer membrane proteins (OMPs), attachment/motility, metabolism, transcription, and translation [5]. Moreover, Eze and co-workers (2018) defined more than 20 genes as the most important in biofilm formation [7]. For example, *bap* is indicated as one of the most important components involved in cell adhesion, which is followed by proper maintenance of *quorum sensing* (QS) [5,7,51,73]; hence, it helps to maintain a stable mature biofilm structure. As was demonstrated, Bap is a part of the type I secretion system (T1SS) [4,71,74]. Furthermore, it probably causes *A. baumannii* to remain viable in diverse environments and protects against antimicrobials and immune cells [7]. Interestingly, it was observed that, in low iron concentrations, increased Bap production affects biofilm formation. What is more, it has not yet been discovered how *A. baumannii* strains can maintain the QS without *bap* genes [75]. This is possibly due to the activity of Bap-like proteins (BLP1 and BLP2) [76]. Another outer membrane exposed protein, Ata, assists in promoting the biofilm formation, adhering to the host tissues by binding to the type IV collagen and other extracellular matrix/basal membrane (ECM/BM) proteins [4,77]. Nevertheless, it was estimated that only 58.6% of tested strains had an *ata* gene within their genome. Expression of *bla*_PER-1_ (*bla* refers to β-lactamase and PER refers to *Pseudomonas* extended resistance, as well as the initials of its discoverers (Patrice, Esthel, and Rogerleads)) helps to increase cell adhesiveness and biofilm formation, but in strains without *bla* gene expression, these processes are not significantly affected [5,78]. Gene clusters of PNAG feature substrate-specific transmembrane transporter activity and can increase biofilm formation, drug resistance, and the production of extracellular matrix [7]. However, the deficiency of PNAG was reported to enhance the resistance to colistin [79]. Genes of *csu* are responsible for biofilm formation on abiotic surfaces and control pili-biogenesis, which participate in the adhesion. Pilus is formed from proteins encoded by a gene cluster known as the *csu* operon, consisting of four subunits: *csuA/B*, *csuA*, *csuB*, and *csuE*. They are involved in archaic chaperone–usher (CU) pathways. Inactivation of the gene encoding the tip protein, *csuE*, leads to the abolishment of the formation of pilus and biofilm [51]. The *csu* operon is regulated by the BfmRS, which, along with AdeRS [80] and GacSA, belongs to a two-component system (TCS) [81]. BfmRS is responsible for biofilm formation and is also involved in the biogenesis of pili, so TCS plays an integral role in the initial adhesion step of biofilm formation [5,7,82]. BfmRS also regulates a K-locus to obtain polysaccharide capsule production, which is also required for biofilm formation. QS molecules are connected with up-regulation in the expression of the *bfmS* and *bfmR* [7]. The *bfmR* gene has attributed functions, such as desiccation tolerance (controlling the expression of oxidative stress response genes) and also factors following nutrient starvation or high osmolarity [4]. BmfRS might also be involved in the contact-dependent growth inhibition system (CDI), controlling cell–cell and cell–environment interactions, allowing the bacterium to adapt to different conditions [83]. Recently, it was estimated that BfmR and BfmS components act as transductors of a light signal within the cell, affecting its’ motility, which reflects the diversity of BmfRS roles inside the bacterial cell [84]. AdeRS decreases biofilm formation and GacSA controls pili synthesis by regulating *csu* operon [4,5]. Genes *abaI* (signal transduction) and *abaR* (AI synthase receptor) are known to be involved in biofilm synthesis and the maintenance of QS [4,7,85]. The acyl homoserine lactone (AHL) molecule has a connection with *abaR* [86]. In the presence of AHL, the expression of Csu pili is increased and biofilm formation is stimulated [5]. OMPs, which include proteins such as CarO and OmpA, are considered to be associated with the transposon-mediated inactivation of *bfmS* in bacteria, resulting in decreased adhesion to biotic and abiotic surfaces [87]. OmpA is a porin that contributes to drug resistance and adhesion to epithelial cells. Furthermore, it mediates bacterial invasion, enables cell membrane integrity, promotes cell death, and contributes to serum resistance and biofilm formation [4,5,7,51,88]. Overproduction of OmpA may contribute to the development of pneumonia and bacteremia, which is associated with higher mortality risk in such individuals [89]. Outer membrane vesicles (OMVs) are involved in secreting OmpA and make it easier to infiltrate the epithelial cells. Generally, OMVs are known to deliver toxins, virulence factors, and other effector molecules to host cells by Gram-negative bacteria [6]. Intriguingly, bacteria also use OMVs as decoys, preventing the infection of phages [90].

An important role in cell-to-cell communication in biofilm structures is attributed to QS, which is a cell density-dependent signaling mechanism that allows bacterial cells to respond to changes in various environmental factors, such as temperature, oxygen level, acidity, and the overall quality of a growth medium, especially nutrient content [1,7]. By utilizing QS, bacteria can coordinate their behavior both with one another and with different species, potentially leading to infections. QS uses signaling molecules known as autoinducers (e.g., AHL). Biofilms are formed by microcolonies when these bacteria reach a certain level. It is considered that it contributes to better access to resources and antibiotic tolerance [1,51] and modulates cell functions such as pathogenicity and motility. It was demonstrated that the limited iron supply may enhance the expression of QS system genes. It acts as a regulator of virulence factors, whose expression is affected by the density of other bacterial strains [51]. Shih and co-workers (2002) demonstrated that damaged QS-related genes are the reason for the formation of thinner biofilms that become sensitive to kanamycin. This also correlates with lower EPS production [91].

QS signal molecules are degraded by AHL lactonases in a process known as *quorum quenching* (QQ), and up-regulation of lactonase expression in *A. baumannii* mutants was demonstrated to result in reduced biofilm formation [92]. Moreover, recombinant engineered QQ lactonases can be used to effectively disrupt biofilm formation, as demonstrated by Chow and co-workers (2014) in *A. baumannii* clinical isolates [93].

### 4.2. Physicochemical Factors Influencing Biofilm Formation by A. baumannii

Environmental factors, such as temperature, osmolarity, ferrous ion concentration, nutrient availability (e.g., glucose), oxygen content, quality of materials on which the biofilm grows, light, and acidic conditions influence biofilm formation [1,7]. Although the lack of oxygen and nutrients induce dispersal of biofilms [1], concentrations of nutrients that are too high might impede its formation [7], as it causes dissolution and minimal competition between strains, which is necessary for its aggregation. The accessibility of supplements such as calcium, phosphate, and sucrose doubles the growth of biofilms [51]. Interestingly, the optimal temperature for a *A. baumannii* biofilm was determined to be 25 °C, which explains why *A. baumannii* is the predominant pathogen in a hospital environment [59]. Another survey conducted on the *A. baumannii* strain ATCC 17978 showed that the biofilm formed better at 28 °C; however, on the other hand, the strain was more mobile at 37 °C (growing faster but producing fewer polysaccharide envelopes) [94]. This suggests that incubation temperature may affect the phenotype of the *A. baumannii* strain ATCC 17978. As it has been shown in the case of *Pseudomonas aeruginosa*, increased gene expression associated with virulence factor production, such as rhamnolipids, is induced at 37 °C. This was also demonstrated to apply to the expression of virulence factors such as OmpA and Paa in *A. baumannii*, as it was greater at 37 °C than at 28 °C. In other research (2021), an optimal temperature of 30 °C with a constant pH of 7.0 supplemented with a sodium chloride medium were indicated to be the most beneficial conditions for the formation of a biofilm [95]. Studies conducted by Gentile and co-workers (2014) showed that *A. baumannii* strains ATCC 17978, ACICU, 50C, and RUH5875 produced more biofilm when grown on iron-containing media [96].

A glucose-based medium can induce the biofilm formation of *A. baumannii* and increase iron uptake. Bacteria adhere rapidly and tightly to positively charged surfaces and by divalent cations [1,7]. Strains of *A. baumannii* have diverse environmental niches in regard to the formation of biofilms. The *Acinetobacter calcoaceticus—Acinetobacter baumannii* complex (ACB complex) produces biofilms at the solid–liquid and air–liquid interfaces. Other ACB complexes produce the highest biofilms at the air–liquid interface. Pellicle leads to biofilm formation when it forms at the liquid–air interface [51], which is indicated to be better than the solid–liquid interface [7]. Disinfectants cause a reduction in biofilm formation [97]. The flow influences the composition and cohesiveness of the EPS matrix. Iron has a strain-dependent association, and, at low concentrations, it triggers the up-regulation of Baps while, at higher concentrations, it allows for increased resistance to antibiotics [73]. Interestingly, it was observed that blue light may also ensure motility and biofilm formation [98].

A formed biofilm facilitates the survival and stability of *A. baumannii* on abiotic hospital surfaces [7]. It was proven that microorganisms prefer to grow on rough surfaces of steel parts of medical equipment [54]. Granularity and irregularities ensure a shield for the bacteria as they protect microorganisms against shear forces, hence facilitating adhesion [51]. Nevertheless, the development of biofilm is not only affected by surface porosity and the aforementioned availability of nutrients but is also influenced by a fluid flow (shear force) and surface charge. Therefore, it has been demonstrated that microorganisms adhere more rapidly to hydrophobic, nonpolar surfaces than to hydrophilic ones [1]. Clinical isolates prefer polycarbonate surfaces to develop biofilm rather than glass, polypropylene, porcelain, and rubber [99], whereas, Hendiani and co-workers (2015) proved that *A. baumannii* more frequently formed biofilm on polypropylene than on polycarbonate surfaces [100]. The ability of *A. baumannii* to form matured biofilms on polypropylene, polystyrene, titanium, and other medical-device-associated materials is attributed to the presence of the earlier-mentioned Bap [7]. Moreover, *A. baumannii* may also persist for days on inanimate surfaces such as pillows, mattresses, bedrails, tables, glass, rubber, porcelain, and sinks [101].

## 5. Strategies to Inhibit Biofilm Development

It is believed that the best strategy for removing bacterial biofilm is to prevent its development in the early stages. To reduce the risk of developing biofilm in industrial settings, complex hygiene procedures should be implemented, generally involving more frequent and thorough cleaning and chemical disinfection of all surfaces [54]. To reduce the nosocomial infection rate, it is critical to remove previously established biofilms, which can be achieved by degrading the extracellular matrix and/or eradicating bacteria inside the structure [55]. Strategies that are used include inhibition of the initial attachment of bacteria to surfaces, rupture of targets of biofilm during the maturation process, and a signal interference approach or QS. The changing of chemicals and the physical properties of biomaterials is a mediator in inhibiting the initial step of biofilm formation. Enzymes that degrade biofilm matrix, surfactants, physical forces, amino acids, free fatty acids, and nitric oxide donors belong to the mediators inhibiting biofilms [102]. Uppu and co-workers (2016) proved that the design of polymers with cationic and hydrophobic domains that interact with the membranes of Gram-negative bacteria, can result in membrane disruption and consequent cell death. Studies showed that an amphiphilic polymer based on maleic anhydride with an amide side chain can disrupt *A. baumannii* biofilms [103]. Moon and co-workers (2017) looked for agents to reduce biofilms formed on medical equipment. Their research showed that trimethoprim–sulfamethoxazole inhibits the expression of *csu* genes involved in pili synthesis by causing folate stress, which disrupts the necessary processes for pili formation [104]. Prebiotic metabolites such as riboflavin, raffinose, citrate, inulin, trehalose, and sorbitol inhibit biofilm growth and formation [1]. A combination therapy of antibiotics may be effective in treating biofilm-associated infections [95]. Another practical approach to inhibiting or disrupting biofilm is targeting AHL—QS autoinducer molecules. Zhang and co-workers (2017) showed that the AHL lactonase, MomL, is responsible for reductions in *A. baumannii* biofilm formation [105]. Moreover, it was proved that the combination of imipenem and silver nanoparticles might also eradicate biofilms [100]. However, one of the most promising possibilities is the application of preparations containing phages or phage-derived enzymes [106].

## 6. Phages and Their Use in Targeted Phage Therapy

Phages are ubiquitous viruses that infect bacteria. Due to their high host specificity, they are one of the most promising alternatives for treating bacterial infections [107]. They were discovered in the 1920s independently by Felix d’Hérelle and Frederick Twort [108]. There are an estimated 10^31^ phage particles on our planet [109]. Therefore, it may be that many species of these viruses remain uncharacterized; as a result, there may still be many unknown viral agents with potential use in treating bacterial infections. Phages can be found in many environments, such as fresh water, sewage, soil, and the microbiota of multicellular Eukaryota [110,111]. Phage morphology is highly varied, but those infecting *A. baumannii* strains are most often complex (tailed) viruses with podovirus, myovirus, or siphovirus morphotypes [108]. During infection, phage particles adsorb to the bacterial cell surface (of their specific host), inject their genetic material, alter the expression of the host genes, and enslave bacterial cell replication machinery to express viral genes [112,113,114,115,116]. Replication of their genome results in the assembly of new virions and, finally, the disruption of the host cell to release progeny virions [12,117,118]. The following features are considered as advantages of phages over the traditional, antibiotic-based approaches: (i) they may be used to target and eliminate pathogenic bacteria at a high level of specificity, (ii) they amplify at the infection site, (iii) bacteria are less likely to develop resistance towards phages, and (iv) phages are generally regarded as safe for eukaryotic cells and mammalian organisms [53,90,119,120,121,122,123,124].

Bacteriophages can adhere to specific receptors (present on the bacterial cell surface) and penetrate biofilm layers using pores and channels, hence destroying the biofilm matrix [121,125]. If receptors become unavailable for bacteriophages (e.g., hidden behind polysaccharide extracellular matrices [90]), viruses are unable to adsorb and infect the host cell [12]. Phage-encoded capsule depolymerases have an affinity to the capsular polysaccharides and function as primary receptors on the bacterial surface [126]. These enzymes allow bacteriophages to break down the defense barrier and interact with host receptors [127], such as capsular polysaccharides, extracellular polysaccharides, OMPs [128], and LPS [129]. These receptors determine a host range of bacteriophages and are recognized by bacteriophage receptor-binding proteins (RBPs), whose composition is varied due to the diversity-generating retroelements (DGRs), enabling mutations within the viral genome and adaptation to their new host by changing the protein composition within RBP structures [90,113,123,130].

One of the first studies based on the antimicrobial activity of phages (phage T4) against the *E. coli* biofilm structure was conducted by Doolittle and co-workers [131]. Since 1995, a lot of research has been focused on the phage activity against these structures [132]. Destroying biofilms with phages can be divided by five main ways: (i) degradation from extracellular layers to intracellular layers (the use of phage-derived endolysin); (ii) degradation from intracellular layers to extracellular layers (classical phage therapy with single phage or phage cocktail); (iii) chemical degradation of EPS (biofilm matrix dispersion; the use of phage-derived depolymerase) [133]; (iv) combination therapy with the use of both phages and antibiotics (phage–antibiotic synergy, PAS) [134]; and (v) genetically engineered bacteriophages with antimicrobial proteins within their genome, enhancing antimicrobial activity [135,136].

Phage therapy depends on the application of targeted phages to infect and kill bacteria that cause infection. The advantages of this therapy include phage amplification at the infection sites and overcoming bacterial resistance via occurring coevolution of bacteria and their bacteriophages. The rate of bacteria acquiring resistance to bacteriophages is lower when compared to the development of resistance to conventional antibiotics [12,51,106]. Potential therapeutic phages are evaluated regarding their biological properties, such as morphology, host range, burst size, and stability under different conditions of temperature, pH, and long-term activity during storage [130]. One of the most important aspects of phage therapy is the optimal titer of phages intended to be applied, since—theoretically—the more phage particles in a therapeutic sample, the faster the treatment. Bagińska and co-workers (2023) showed that the optimal MOI varies even within phages infecting the same host and that some viruses prefer lower MOIs (lower phage density, therefore lower competition for the receptors) [137]. Sometimes, different values of MOI give the same results of host lysis kinetics [53]. The killing curve is used to evaluate the lytic activity and efficacy of phages in controlling the growth of *A. baumannii* at different MOIs [10,138]. Nonetheless, it is important to mention that phages intended for therapeutic applications should not contain any virulence (e.g., bacterial toxins) and/or antibiotic resistance genes within their genome [12,139], as, if they possess such determinants, they could serve as vectors for HGT and, upon infection of bacterial cells, equip their host with mechanisms that contribute to their pathogenicity [140,141,142]. Therefore, before application in phage therapy, phage genomes have to be sequenced and carefully checked with the use of bioinformatic tools for any unfavorable elements (so-called moderate phage markers) [143]. Finally, purified phages whose strictly lytic strategy of replication has been confirmed can be introduced to the phage therapy.

Currently, the available literature data indicate that MDR bacterial infections can be successfully treated with lytic phages [53,55,90,119,120,121,122,144,145,146,147,148,149,150,151,152,153]. However, there is still more data needed about case studies documenting the successful treatment of bacterial infections with phages. The diversity among phages, their hosts, and patients, is yet too overwhelming to define that these viruses are a utilitarian tool in fighting bacterial infections.

Phages are good candidates for therapeutic purposes and should ideally have, among other properties a fast adsorption rate, broad host range, high depolymerizing activity, and antibacterial activity against both planktonic and biofilm-associated bacteria [10]. The possible synergy between phages and antibiotics (PAS) may lead to accelerated bacterial cell lysis, enhanced phage burst size, as well as resensitization of pathogens to certain antibiotics [10,121,154]. In the case of *A. baumannii* specifically, Wang and co-workers (2021) observed that bacteria resistant to myophage Phab24 were characterized by increased sensitivity to colistin even though, on the genetic level, the antibiotic resistance mechanism remained unchanged [155]. However, phage therapy combined with the use of antibiotics is not solely prevalent in treating infections caused by *A. baumannii* [156,157,158,159,160,161,162]. Although synergy usually occurs, antagonism has also been reported and was reviewed by Łusiak-Szelachowska and co-workers [121].

## 7. Bacteriophages’ Activity Against Biofilm-Forming by *Acinetobacter baumannii*

Initially, bacterial biofilm was eradicated with antibiotics (single or mixed chemotherapeutic agents) [163]. It has been observed that the combination of two drugs can show synergistic effects, thus surpassing the effect of single-drug antibiotic therapy. However, the mechanisms of action of individual drugs should always be considered when a multi-drug regimen is implemented, as some combinations may also act antagonistically, exerting an opposite effect [12]. In addition, high antibiotic dosage may lead to the development of antimicrobial resistance [10]. Dijkshoorn and co-workers (1996) conducted their research based on the effectiveness of mixed drugs, e.g., colistin–levofloxacin, colistin–tigecycline, and tigecycline–levofloxacin. The combination of colistin with levofloxacin showed the strongest antibacterial activity [164]. For biofilms of CRAB or carbapenem-susceptible strains, the combination of sulbactam–tigecycline may be also applied. Clarithromycin, a macrolide antibiotic, can be used with colistin, tigecycline, or imipenem to reduce the infection [52].

Nevertheless, since bacteria gain resistance to many classes of antibiotics, new methods for combating biofilms are needed [163]. Therefore, one of the most promising biocontrol agents is bacteriophages, which can undergo evolution and adjust to their host [86].

Nevertheless, bacteria are also able to evolve and acquire resistance mechanisms. The most interesting resistance mechanism in the subject matter covered is the clustered regularly interspaced short palindromic repeats (CRISPR)-Cas system [165]. It allows bacteria to gain resistance to specific fragments of phage DNA by “remembering” them within libraries of similar fragments flanked by protospacer adjacent motif (PAM) sequences [166]. Interestingly, this so-called “bacterial immune system” [167] can be stimulated with QS [168]. It was also proven that QS could help the bacteria recruit many CRISPR-Cas systems simultaneously, increasing bacterial resistance to bacteriophages [169]. Another mechanism that allows *A. baumannii* to resist spreading bacterial viruses within its population hinges on the mechanism of abortive infection (Abi), especially on the toxin–antitoxin (T/AT) system [90]. It is thought that this pathogen “uses” the viral genome as a provocation to repress the antitoxin promoter or to terminate its transcription, which is followed by an increased concentration of the toxin, resulting in host cell death [170,171]. Hence, these dead cells might not only be a brilliant source of EPS components [12] but also genes that can be acquired by HGT (especially the transformation process), strengthening persister bacteria within the biofilm structure [64]. Although the “bacterial fortress” is thrillingly prepared, phages (with many counter mechanisms acquired from the mentioned co-host evolution [90,172,173]) are one of the most promising agents helping us fight these structures on medical equipment, even in terms of polymicrobial biofilms [138].

Morris and co-workers (2019) showed that phages have anti-biofilm activity on the abiotic, titanium surfaces of orthopedic equipment (research was conducted on *S. aureus*) [174]. Furthermore, the anti-biofilm activity of phages was proven in other pathogens beyond *A. baumannii*, including MDR bacterial pathogens from the ESKAPE group, such as *Enterococcus faecalis* [175], *S. aureus* [176], *K. pneumoniae* [177], *P. aeruginosa* [178], and *Enterobacter cloacae* [179]. Phage activity against biofilm formation by *A. baumannii* was quite extensively studied [10,53,106,134,180,181,182,183,184,185,186,187,188,189,190,191,192,193,194,195,196]—the most important examples are gathered in Table 2 and Table 3.

## 8. Anti-Biofilm Activity of Phage-Derived Enzymes

Endolysins, phage-encoded hydrolases, are a class of potential antimicrobials for the treatment of drug-resistant bacterial infections. They are lytic enzymes produced during the last step of the bacteriophage replicative cycle and act by disrupting the integrity of bacterial cell wall from within, which results in a sudden drop in osmotic pressure, leading to bacterial cell lysis, making it possible for phage progenies to be released from the host and into the environment [116,197,198,199]. This mode of action also makes lysins promising tools for fighting bacterial infections. Compared to traditional broad-spectrum antibiotics, they exhibit high specificity at bacterial genus or species level without interacting with the surrounding microbial cells. Furthermore, they also exhibit synergistic activity with different antibacterial agents, reduce the probability of inducing resistance, and are effective in biofilms. Based on their mode of action on the bonds in the peptidoglycan layer, endolysins are divided into five categories: (i) N-acetyl-β-D-intracellular amidase; (ii) N-acetyl-β-D-glucosaminidase; (iii) transglycosidase; (iv) N-acetyl-leucoyl-l-alanine amidase; and (v) L-alaninoyl-D-glutamate endopeptidase [199]. While the mentioned enzymes are a potent tool against Gram-positive bacteria, in the case of Gram-negative pathogens, their use is limited by the OM, which prevents exogenously applied endolysin from accessing the PG layer. The use of chelators (ethylenediaminetetraacetic acid, EDTA), weak organic acids, or high hydrostatic pressure has been suggested for increasing OM permeability. The combination of endolysin–EDTA may, however, be limited only to the topical treatment of localized bacterial infections, such as burn wounds or eye and ear infections [197]. Interestingly, recent studies on the novel endolysins LysAm24, LysAp22, LysECD7, and LysSi3 [200,201], which are active against Gram-negative ESKAPE pathogens, indicate that these enzymes can demonstrate effective bactericidal action without additional membrane permeabilization, although the addition of EDTA enhanced their activity under neutral and alkaline conditions, under which their bacteriolytic effect was otherwise somewhat diminished. Moreover, these endolysins were tested and proved effective against a range of Gram-negative pathogens—roughly 60 to over 80 out of 120 MDR isolates representing *A. baumannii*, *P. aeruginosa*, *K. pneumoniae*, *E. coli*, *Salmonella enterica*, *Enterobacter* spp., and *Campylobacter jejuni* [202], revealing a much broader spectrum compared to their progenitor phages. While these experimental data seem to be in contrast with the generally accepted high specificity of phage lysins, the authors argue that the conserved structure of the peptidoglycan of Gram-negative bacteria may contribute to the breadth of action of the enzymes targeting them [201].

Biofilms may develop within water dispensers in poultry production systems, highlighting the potential use of depolymerase as a management practice to disrupt biofilms and potentially reduce antimicrobial resistance. The supplementation of microbial muramidase with endolysin activity positively affects growth and gastrointestinal functionality by the reduction in antimicrobial resistance in poulters, which are a reservoir of MDR organisms [199].

Among the phage-encoded depolymerases, peptidoglycan hydrolases, endorhamnosidases, alginate lyases, endosialidases, and hyaluronate lyases are present. Enzymes can occur in two forms: as an element of a virion particle, predominantly in the form of tail spikes or fiber proteins mainly attached to the base plate, and as a soluble protein released during host lysis after phage maturation [121,203]. The mentioned enzymes partially break down the bacterial cell wall, impacting structural components like LPS, PG, and CPSs, including EPS [53,121,198]. CPSs cover the bacterial surface with layers so that they increase antimicrobial resistance and evasion of host immune defenses and also promote survival and adaptation to various environmental conditions [55,130,204]. Tail spike protein (TSP) removes CPSs, causing a loss of integrity in the bacterial cell envelope. This exposure makes the pathogen more vulnerable to immune attacks, resulting in decreased bacterial infectivity [55]. Enzymes that degrade EPS also facilitate phage penetration in biofilm [121]. EPS-degrading enzymes might also increase the diffusion of antimicrobial particles (antibiotics and/or phages), which may result in the more effective treatment of bacterial infections [199]. Therefore, they may facilitate the elimination of biofilms during bacterial infections by proper phage recognition and adsorption to its host [12,53]. Moreover, TSP is also able to inhibit biofilm formation [55]. It is suggested that multiple depolymerases are needed for treating mixed biofilms [121]. Because of these activities, the enzymes can be implemented as alternative antibacterial therapies [130]. In the treatment of bacterial infections caused by planktonic forms or biofilm-forming strains, phage polysaccharide depolymerases (in vitro, the expression and activity of these proteins is observed in the form of a halo surrounding phage plaques [205]) can be used in combination with antibiotics [206,207,208]. Phage-derived enzymes active against *A. baumannii* biofilm are presented in Table 4.

An interesting paper was published in 2022 that proposed the use of phage-encoded depolymerase with colistin as an adjuvant against biofilms formed by *A. baumannii* [217]. Firstly, three concentrations of the Dpo71 depolymerase (1, 10, and 40 µg/mL) were tested on a mature biofilm of *A. baumannii* MDR-AB2. The lowest concentration did not cause a significant decrease in the percentage of biofilm biomass (~10%). However, concentrations of 10 and 40 µg/mL showed better inhibition of biofilm formation (~40% decrease in the biofilm biomass). In order to further improve the anti-biofilm activity of the preparation, the synergy between Dpo71 (10 µg/mL) and colistin (4 µg/mL) was investigated. The inhibition of biofilm formation by colistin alone was slightly more potent than that of Dpo71 (~55%), and the synergy between Dpo71 and the antibiotic showed a significant improvement, i.e., a ~75% decrease in biofilm biomass. Therefore, these data strongly indicate that phage-encoded depolymerases and colistin can complement their respective mechanisms of action and act together to inhibit the biofilm of *A baumannii* significantly better than each of them acting alone.

## 9. Alternative Treatments for Infections Caused by *A. baumannii* Biofilm

Antibiotic-based antimicrobial therapy may induce different side effects, such as toxicity, microbiota disturbance, and the acquisition of resistance mechanisms observed in bacteria [12]. On top of that, as mentioned before, *A. baumannii* isolates are often characterized by remarkable genomic flexibility, which allows them to acquire resistance mechanisms to many antibiotics, even from other bacterial species [27].

For both prevention and disruption of biofilms, physical, chemical, and biological methods are used, such as surfaces coated with bacteriocins or essential oils, as well as small molecules that interfere with the expression of virulence genes responsible for biofilm formation [55]. Natural products, such as microbial, plant-based (essential oil from e.g., *Salvia glutinosa*), and animal-derived products (antimicrobial peptides from blowfly maggots *Calliphora vicina*), are effective at treating infections [7]. Bulgecin A, a natural derivative of *Pseudomonas mesoacidophila*, acts as a lytic transglycosylase inhibitor and operates synergistically with β-lactams. This compound may act as a booster to enhance the efficiency of carbapenems against *A. baumannii* infections. Farnesol is another example which is a natural product derived from *Candida albicans* applied to disrupt QS, deforming the integrity of the cell membrane of *A. baumannii*, changing the cellular morphology, and hence enhancing the sensitivity of MDR *A. baumannii* strains to colistin [51].

Another interesting example of using the antimicrobial capabilities of plants involves terpenes derived from oregano essential oil (OEO), such as carvacrol and thymol. Their antibacterial properties stem from the presence of a hydroxyl group in their structure [218]. Tapia-Rodriguez and co-workers (2023) showed that terpenes from OEO have an inhibiting effect on *A. baumannii* biofilm and also interfere with synthesis and the assembly of pili, destroying the existing type IV pili, which participate in motility, adhesion, and biofilm formation [219]. In addition to the antibacterial effects of carvacrol and terpenol on *A. baumannii*, they have been shown to also affect other pathogenic bacteria, causing the destruction of membrane proteins and the lipids of the bacterial cell wall, inhibiting DNA synthesis, silencing efflux pumps, and interfering with proper enzyme activity [220].

AMPs may be derived from plants, animals, insects, protozoa, and fungi [4,221], and they may also be synthetically produced [222]. In animals, including humans, they represent elements of the innate humoral immune response [12] and belong to the first line of defense against invading pathogens. AMPs are small, amphipathic, hydrophobic, or hydrophilic, and they are typically cationic [4] and evolutionarily conserved [222]. Natural AMPs consist of 12–50 amino acids and usually contain lots of arginine and lysine residues [223]. Due to their cationic character, they can interact with the negative charge on the membrane of Gram-negative bacteria. It was demonstrated that, compared to antibiotics, AMPs have reduced immunogenicity, better tissue penetration, and relatively low manufacturing costs, which are clear advantages. Peptide length, encapsulation, and composition can be altered through peptide modification to enhance the target specificity of AMPs [4]. De la Fuente-Núñez and co-workers (2014) demonstrated that low concentrations of synthetic peptides designated as innate defense regulators (IDRs) 1018 [224], whose sequence is based on bovine host-defense peptides (HDPs), a derivative of the bactenecin Bac2A [225], caused the *A. baumannii* biofilm to disperse, while higher doses triggered biofilm cell death.

Different substances, such as hydrogen peroxide and hydrogen peroxide-based formulations, silver nanoparticles, and cyclic di-GMP, can cause the abolishment of biofilm as well [7]. Nitric oxide (NO) has antimicrobial activity as well as is immunomodulatory properties, regulating wound healing. Interestingly, NO-releasing nanoparticles (NO-NPs), which were formed by using nanotechnology-based silane hydrogel, were able to reduce collagen degradation by bacterial collagenases and accelerate wound healing [226,227]. Also, gallium, a metallic element, can prevent biofilm formation [228,229,230]. Gallium is used in the form of simple inorganic and organic salts and in the form of complexes of organic compounds, such as protoporphyrin IX. Ga^3+^ shows structural similarity to Fe^3+^ and therefore makes similar coordination bonds to iron [229]. For this reason, Gallium can compete for the active site in iron-containing enzymes as well as in transferrin, lactoferrin, and siderophores; however, unlike iron, it is not reduced. In addition, if it replaces iron in redox enzymes, essential reactions related to, for example, DNA synthesis are inhibited [231]. Nanoparticles consist of metals such as silver, gold, platinum, and zinc [232]. Silver nanoparticles (AgNPs) penetrate the bacterial cell membrane, releasing silver cations that disrupt electron transport and essential signaling pathways and produce reactive oxygen species (ROS), which damage key cellular components such as DNA, cell wall structures, and proteins. This process allows bacteria to evade antimicrobial mechanisms. Depending on their concentration, AgNPs can simultaneously induce apoptosis and inhibit the synthesis of new DNA molecules [12,51]. Nanoparticles with chitosan, a non-toxic biodegradable polymer, either alone or in combination with antibiotics, are also used [233]. Nanoparticles are effective in the treatment of complex skin infections [227]. The attachment of silver nanoparticles is effective against several MDR pathogens (for example, *Enterobacter* spp., *Acinetobacter* spp., and *Klebsiella* spp.) and decreases their biofilm-forming activity [234]. AgNPs disturb bacterial growth and proliferation and cause the downregulation of virulent and biofilm-related genes at the transcriptional level. AgNPs inhibit *A. baumannii* growth and prevent colonization and biofilm formation on the human lung epithelia [51]. Another example of an alternative antimicrobial strategy is iron chelation therapy, as iron acts as a cofactor in many bacterial cellular processes [235]. Thus, iron chelators and iron competitors are potential antimicrobial agents [236], interfering with iron acquisition by bacterial siderophores. Ran and co-workers (2021) constructed a unique cationic photosensitizer (Nile Blue Dyes) and bacteriophage-based photodynamic (ABP) antimicrobial agent (APNB) for photodynamic antimicrobial chemotherapy (PACT) against multidrug-resistant *A. baumannii* [237]. It can produce ROS. APNB can target bacteria and may be used in combination therapy (i.e., PACT). PACT is involved in photooxidative stress [12]. PACT agents have a high selectivity, and sensitivity. PACT can act both on Gram-positive and Gram-negative bacteria; it has low toxic and side effects and the ability to eliminate drug resistance [237]. It uses many inorganic and metallic nanocomposites. Unfortunately, nanomaterials are difficult to degrade, but organic small molecules have an easy metabolism and high biosafety, which is why they are a good to use in PACT therapy. However, their disadvantage is inefficient production of ROS, which results in a poor ability to kill bacteria. Photodynamic inactivation (PDI) is used for MDR bacterial infections in order to heal. The addition of biological or chemical molecules can be used for the purpose of enhancing the effectiveness of photosensitization by anionic photosensitizers such as erythrosine B [238]. The addition of this molecule leads to changes in molecular charge and the structure of photosensitizer; additionally, it also modifies the native consistence of the OM/EPS [239]. It was proven that PDI that uses erythrosine B in acetic acid or chitosan is very effective against *A. baumannii*-based planktonic cells and can eliminate them. Acetic acid is responsible for modifying the molecular charge of photosensitizers, whereas chitosan destabilize the biofilm architecture [240,241]. PDI causes the fast killing of bacteria and is targeted at the broad-spectrum of microorganisms, independent of their antimicrobial resistance profiles [242]. PDI is a combination of a non-toxic dye known as photosensitizer (PS) and visible light. It produces cytotoxic ROS, which can cause damage cellular components such as DNA, membrane lipids and proteins, finally leading to cell death. When a photosensitizer molecule has an anionic charge, it cannot interact with Gram-negative bacteria [206]. It is caused by an bacterial OM barrier that prevents the uptake of anionic compounds. Also, negatively charged EPS, which surrounds and protects cells, restricts penetration of anionic photosensitizer into the biofilm. Gram-positive bacteria are sensitive to photodynamic inactivation with erythrosine because they have a permeable cell wall. They have a limitation in terms of the penetration of photosensitizers. In contrast, Gram-negative bacteria are less vulnerable to photodynamic inactivation with erythrosine. They contain an outer membrane which makes a barrier between the cell and its environment. Thus, they create a problem in penetrating PS. The outer membrane of Gram-negative bacteria has a negative surface charge, thus is impermeable to anionic compounds. Acetic acid increases penetration into the outer membrane of Gram-negative bacteria by means of PS, which bears an anionic charge, such as EB. Use of acetic acid is not enough to cause lethal effect on *A. baumannii* biofilm cells. Chitosan helps PDI to act on the cells that are released from the biofilm, permeabilizes the outer membrane of Gram-negative bacteria, and, additionally, acts as a drug carrier for the delivery of erythrosine B to biofilm [239].

Adjuvants in combination with antibiotics may be appropriate for use in clinical antibacterial practice [243]. Such an approach increases antibiotic intake through bacterial membranes, inhibits efflux pumps, and changes the physiology of resistant cells, causing the inhibition of resistance mechanisms.

Prophylactic vaccination is another approach to combating bacterial infections. Outer membrane proteins, for example OmpA, the most promising antigen, are used in vaccine development [12].

Recently, a few new antibiotics have been discovered, for example, the GSK-3342830 injectable cephem that targets Gram-negative bacteria and demonstrates activity against CRAB [244]. Another example is an already-mentioned novel cephalosporin-based antibiotic cefiderocol (S-649266) that is cephalosporin-conjugated with a catechol siderophore on its side chain, which also exhibits activity against CRAB [245,246]. Further, a natural siderophore fimsbactin and a combination of sulbactam and durlobactam act as a novel beta-lactamase inhibitor [51,246,247].

Probiotics, i.e., live microorganisms that are administered to the host to ensure health benefits, protect the host against *A. baumannii* pathogenicity [248]. Immunomodulators, such as lysophosphatidylocholine can decrease the severity of infections when combined with antibiotics as well as probiotics [249]. Phages containing the integrase gene may be used as a viral vector-mediated gene therapy due to their ability to integrate plasmid DNA into the host’s chromosome. Consequently, temperate phages, which are described by Soontarach and co-workers (2022), may be candidates for effectively combating infections caused by *A. baumannii* [130].

**Figure 2:**
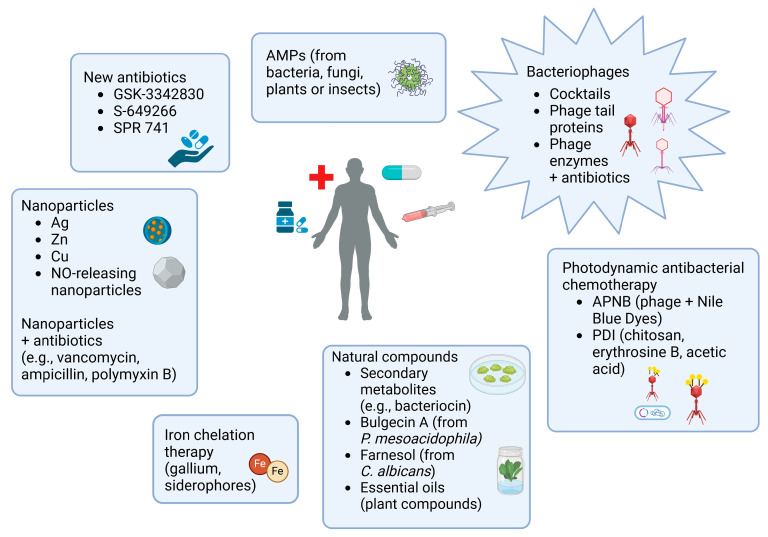
Future therapies targeting *A. baumannii* infections (BioRender, agreement number: NK27IRP7SL).

Future therapies against *A. baumannii* are shown in Figure 2.

## 10. Conclusions and Perspective

Bacteriophage therapy presents a promising and targeted solution for addressing the challenge of *Acinetobacter baumannii* biofilm-associated infections, especially in the context of increasing global antibiotic resistance. Phages have a unique ability to specifically target both planktonic bacterial cells and biofilm structures and are amplified at the infection site, which makes them an alternative or complement to traditional antibiotic treatments. Although experimental data show encouraging results, several challenges still need to be overcome, including phage resistance, strain specificity, and difficulties in large-scale application and regulation.

Studies on bacteriophage application in treating biofilm-associated infections caused by *A. baumannii* are promising, yet they have several weaknesses and limitations. Most of the studies are conducted in vitro and therefore do not provide comprehensive and reliable results that can be readily translated into the clinic, where factors such as the immune system, tissue interactions, and phage dynamics (e.g., the ability to penetrate biofilm structures) at the site of infection need to be considered.

The development of genetically engineered or synthetic phages could significantly enhance their effectiveness and reduce the risk of acquired resistance. The use of phage cocktails and combined therapies with antibiotics also hold great potential for improving treatment outcomes. Therefore, further research on phage–host interactions, biofilm penetration, and the optimization of phage therapy is essential. In particular, rigorous clinical trials are required to evaluate safety, efficacy, and the long-term effects of phage-based therapies.

In the near future, integrating phage therapy into clinical practice could offer an innovative, sustainable solution to combat MDR infections, particularly those caused by *A. baumannii*. With ongoing advancements, bacteriophages may soon play a central role in overcoming one of the most pressing challenges in modern medicine: biofilm-related antimicrobial resistance.

## Figures and Tables

**Table 1:** Factors responsible for biofilm formation in *A. baumannii* strains and their possible functions.

The Factor Responsible for Biofilm Formation in *A. baumannii*	Function	Reference
OMPs (including OmpA, OmpW and CarO)	Contributes to drug resistance, adhesion to epithelial cells, mediates their invasion, enables cell membrane integrity, promotes cell death, contributes to serum resistance, biofilm formation, transposon-mediated inactivation of *bfmS* in bacteria, regulates iron uptake	[4,5,7,51]
Bap	Involved in resistance mechanisms, and required for biofilm reduction, maintains a stable mature biofilm structure, mediates adherence to bronchial cells, interbacterial cell adhesion, enhances hydrophobicity, structural integrity as well as water channel formation, up-regulation is linked to low iron concentration	[5,7,51]
Bla(PER-1)	Increases cell adhesiveness and biofilm formation	[5]
Ata	Assists in biofilm adherence to the host	[4]
PNAG (and its *pgaABCD* gene)	Substrate-specific transmembrane transporter, increases biofilm formation, enhances drug resistance, protection against innate host defense, cell–cell adherence, and production of the extracellular matrix	[1,7]
K1 capsule	Mediates resistance to cationic AMPs and serum; K1 locus regulates the production, modification, and export of capsular polysaccharides	[1,25]
Csu (ABCDE)	Biofilm formation on abiotic surfaces, controls pili biogenesis	[51]
Two-component systems (BfmRS, AdeRS, and GacSA)	Biofilm formation, involved in biogenesis of pili, regulates *csu* operon (including motility) and K-locus to obtain capsule production, regulates *quorum sensing* (QS), amino acid metabolism, resistance to human serum, involved in tolerance to desiccation	[4,5,7,51]
Efflux pump (5 families)	Overexpression leads to multidrug resistance, decreased biofilm production, synthesis and transport of autoinducer molecules, and altered membrane composition	[7]
AbaIR (and *aba* genes)	Regulates the QS system, involved in reducing biofilm formation	[5,7]
AHL	Increases expression of Csu pili and stimulates biofilm formation	[5]
BlsA	Influences virulence through iron metabolism via direct interactions with Fur	[4]
T1SS, T2SS	Maintains biofilm stability, exports Bap beyond the OM, involved in virulence of *A. baumannii*, responsible for secretion of extracellular enzymes—lipases, such as lipoyl synthases LipA, LipH, and proteases, such as CpaA	[4,71]

**Table 2:** Bacteriophage activity against biofilm-forming by *Acinetobacter baumannii*.

Phage Symbol (Morphotype/Type of Replication Cycle)	Bacterial Strain(s) Used in the Experiment	Type of Experiment (In Vitro/In Vivo)/Tested Surface	The Main Result(s) of the Experiment	Reference
vB_AbaP_WU2001 (P/lytic)	ABPW052	in vitro/96-well plate/abiotic	A 48.72% inhibition of biofilm formation, and 78.82% degradation of mature biofilm at 10^8^ PFU/well	[10]
vB_AbaM_ISTD (M/lytic)	6077/12	in vitro/porous glass beads/abiotic	A 30% degradation of mature biofilm at MOI 100	[53]
vB_AbaM_NOVI (M/lytic)		in vitro/porous glass beads/abiotic	A 30% degradation of mature biofilm at MOI 100	
vB_AbaP_B3 (P/lytic)	Ab404_GEIH-2010	in vitro/96-well plate/abiotic	A high degradation of mature biofilm at MOI 10	[106]
	Ab019_GEIH-2010	in vitro/96-well plate/abiotic	A low degradation of mature biofilm at MOI 10	
	Ab034_GEIH-2010	in vitro/96-well plate/abiotic	A high degradation of mature biofilm at MOI 10	
	Ab007_GEIH-2010	in vitro/96-well plate/abiotic	A low degradation of mature biofilm at MOI 10	
	Ab008_GEIH-2010	in vitro/96-well plate/abiotic	A high degradation of mature biofilm at MOI 10	
	NIPH2061	in vitro/96-well plate/abiotic	A high degradation of mature biofilm at MOI 10	
Ab105-2phiΔCI404ad (S/created lytic mutant)	Ab404_GEIH-2010	in vitro/96-well plate/abiotic	A high degradation of mature biofilm at MOI 10	
	Ab019_GEIH-2010	in vitro/96-well plate/abiotic	A low degradation of mature biofilm at MOI 10	
	Ab034_GEIH-2010	in vitro/96-well plate/abiotic	A high degradation of mature biofilm at MOI 10	
	Ab007_GEIH-2010	in vitro/96-well plate/abiotic	A low degradation of mature biofilm at MOI 10	
	Ab008_GEIH-2010	in vitro/96-well plate/abiotic	A high degradation of mature biofilm at MOI 10	
	NIPH2061	in vitro/96-well plate/abiotic	A high degradation of mature biofilm at MOI 10	
vB_AbaP_B3 (P/lytic) and Ab105-2phiΔCI404ad (S/created lytic mutant)	Ab404_GEIH-2010	in vitro/96-well plate/abiotic	A high degradation of mature biofilm at MOI 10	
	Ab019_GEIH-2010	in vitro/96-well plate/abiotic	A low degradation of mature biofilm at MOI 10	
	Ab034_GEIH-2010	in vitro/96-well plate/abiotic	A high degradation of mature biofilm at MOI 10	
	Ab007_GEIH-2010	in vitro/96-well plate/abiotic	A low degradation of mature biofilm at MOI 10	
	Ab008_GEIH-2010	in vitro/96-well plate/abiotic	A high degradation of mature biofilm at MOI 10	
	NIPH2061	in vitro/96-well plate/abiotic	A high degradation of mature biofilm at MOI 10	
AB7-IBB2 (P/lytic)	AIIMS 7	in vitro/96-well plate/abiotic	An 80% inhibition of biofilm formation at MOIs 1000 and 100,000	[181]
AB7-IBB1 (S/lytic)	AIIMS 7	in vitro/human embryonic kidney 293 cell line/biotic	A 50% inhibition of biofilm formation at all tested MOIs (i.e., 0.1, 10, 100, and 1000)	[182]
		in vitro/polystyrene/abiotic	A 75% inhibition of biofilm formation at MOI 100,000	
vB_AbaM_IME_AB2 (M/lytic)	MDR-AB2	in vitro/96-well plate/abiotic	An 88.5% degradation of mature biofilm at MOI 0.1	[183]
		in vitro/metal surfaces (with protocol mimicking clinical settings; European standards EN 13727:2012)/abiotic	A 93.33% degradation of mature biofilm at MOI 10	
AB1801 (S/lytic)	NPRC AB11	in vitro/96-well plate/abiotic	A 66% inhibition of biofilm formation, and a 70% degradation of performed biofilm at MOI 1	[184]
Abp9 (M/lytic)	ABZY9	in vitro/96-well plate/abiotic	A 72.22% degradation of mature biofilm at MOI 10	[185]
Aba-1 (nd/lytic)	AB3	in vitro/96-well plate/abiotic	A 77.69% inhibition of biofilm formation at MOI 0.5	[186]
		in vitro/urine model—standing for the natural environment within the human’s urinary tract/biotic	A 67.45% inhibition of biofilm formation at MOI 0.5	
Aba-2 (nd/lytic)	AB8	in vitro/96-well plate/abiotic	A 55% inhibition of biofilm formation at MOI 0.5	
		in vitro/urine model—standing for the natural environment within the human’s urinary tract/biotic	A 34% inhibition of biofilm formation at MOI 0.5	
Aba-3 (nd/lytic)	AB14	in vitro/96-well plate/abiotic	A 53% inhibition of biofilm formation at MOI 0.5	
		in vitro/urine model—standing for the natural environment within the human’s urinary tract/biotic	A 44% inhibition of biofilm formation at MOI 0.5	
Aba-4 (nd/lytic)	AB23	in vitro/96-well plate/abiotic	A 61% inhibition of biofilm formation at MOI 0.5	
		in vitro/urine model—standing for the natural environment within the human’s urinary tract/biotic	A 52% inhibition of biofilm formation at MOI 0.5	
Aba-5 (nd/lytic)	AB11	in vitro/96-well plate/abiotic	A 35% inhibition of biofilm formation at MOI 0.5	
		in vitro/urine model—standing for the natural environment within the human’s urinary tract/biotic	A 35% inhibition of biofilm formation at MOI 0.5	
Aba-6 (nd/lytic)	AB10	in vitro/96-well plate/abiotic	A 62% inhibition of biofilm formation at MOI 0.5	
		in vitro/urine model—standing for the natural environment within the human’s urinary tract/biotic	A 91% inhibition of biofilm formation at MOI 0.5	
vWUPSU (M/lytic)	NPRCOE 160519	in vitro/96-well plate/abiotic	A 68.3% inhibition of biofilm formation, and 53.3% degradation of mature biofilm at 10^8^ PFU/well	[187]
vB_AbaP_HB01 (P/lytic)	24 different strains: C1, C3, C5, C7, C10, C18, C19, C22, A1, A4, A7, A8, SU1, SU2, SU3, ATCC, M1, M2, M6, M7, M20, M24, M27, M34	in vitro/96-well plate/abiotic	After 6 h—degradation of mature biofilm at the level of 40–86%; after 24 h—degradation of mature biofilm at the level of 18–84%; after 48 h—degradation of mature biofilm at the level of 1–81% among all tested strains	[188]
vB_AbaM_HB02 (M/lytic)		in vitro/96-well plate/abiotic	After 6 h—degradation of mature biofilm at the level of 35–85%; after 24 h—degradation of mature biofilm at the level of 26–85%; after 48 h—degradation of mature biofilm at the level of 1–80% among all tested strains	
vB_AbaP_HB01 (P/lytic)	78 different strains	in vitro/biofilm cultured on a catheter/abiotic	A ~68% inhibition of biofilm formation, and ~78.57% degradation of mature biofilm	[189]
vB_AbaM_HB02 (M/lytic)		in vitro/biofilm cultured on a catheter/abiotic	A ~60% inhibition of biofilm formation, and ~71.43% degradation of mature biofilm	
vB_AbaP_HB01 (P/lytic) and vB_AbaM_HB02 (M/lytic)		in vitro/96-well plate/abiotic	A ~76% inhibition of biofilm formation, and ~83.33% degradation of mature biofilm	
vB_AbaS_SA1 (S/temperate); vB_AbaS_Ftm (S/temperate); vB_AbaS_Eva (S/temperate); vB_AbaS_Gln (S/temperate)	30 different XDR strains	in vitro/96-well plate/abiotic	A ~78% inhibition of biofilm formation, and ~66% degradation of mature biofilm at MOI 1000	[190]

Abbreviations: P—podovirus; M—myovirus; S—siphovirus; nd—no data.

**Table 3:** The activity of single and/or phage cocktail combined with antibiotics against *A. baumannii* biofilm.

Phage(s) Symbol (Morphotype/Type of Replication Cycle)	Tested Antibiotic(s)	Bacterial Strain(s) Used in the Experiment	Type of Experiment (In Vitro/In Vivo)/Tested Surface/PAS and/or Phage Cocktail	The Main Result(s) of the Experiment	Reference
pB3074 (nd/lytic)	cefotaxime (2 × MIC) or meropenem (0.5 × MIC)	Bm3074	in vitro/96-well plate/abiotic	The highest degradation of mature biofilm was observed with phage and meropenem	[134]
Aba-1(nd/lytic); Aba-2 (nd/lytic); Aba-3 (nd/lytic); Aba-4 (nd/lytic); Aba-6 (nd/lytic)	amikacin, gentamicin, tobramycin, colistin, imipenem, meropenem, trimethoprim/sulfamethoxazole, ciprofloxacin, levofloxacin	AB20	in vitro/urine model—standing for the natural environment within the human’s urinary tract/biotic/phage cocktail and PAS	A 98.6% biofilm degradation considering phages (10^7^ PFU/mL) and 0.5 × MIC trimethoprim/sulfamethoxazole	[186]
vB_AbaP_HB01 (P/lytic) and vB_AbaM_HB02 (M/lytic)	colistin	24 different strains	in vitro/96-well plate/abiotic	After 6 h—degradation of mature biofilm at the level of 42–87%; after 24 h—degradation of mature biofilm at the level of 28–85%; after 48 h—degradation of mature biofilm at the level of 0–81% among all tested strains	[188]
φAB182 (M/lytic)	ceftazidime, polymyxin B, cefotaxime, colistin	MDR strains	in vitro/96-well plate/ abiotic/PAS	The highest degradation of mature biofilm with combining phages and colistin, followed by polymixin B, ceftazidime, and cefotaxime	[192]
T1245 (P/lytic)	imipenem or colistin, meropenem or ceftazidime	MDR strains	in vitro/96-well plate/ abiotic/PAS	An ~80% degradation of mature biofilm; synergistic effect of phage in combination with all tested antibiotics was observed	[193]
vB_AbaM-IME-AB2 (M/lytic)	colistin (2 × MIC)	MDR-AB2	in vitro/96-well plate/abiotic/first phage then colistin	A ~90% degradation of mature biofilm	[196]
			in vitro/96-well plate/abiotic/first colistin then phage	A ~72% degradation of mature biofilm	
			in vitro/96-well plate/abiotic/colistin and phage simultaneously	A ~88% degradation of mature biofilm	
vB_AbaS_SA1 (S/temperate); vB_AbaS_Ftm (S/temperate); vB_AbaS_Eva (S/temperate); vB_AbaS_Gln (S/temperate)	ampicillin/sulbactam	30 different XDR strains	in vitro/96-well plate/ abiotic/PAS	At 10×, 5×, and 1 × MIC, the PAS effect on biofilm degradation was observed	[190]
	meropenem			At 1 × MIC, the PAS effect on biofilm degradation was observed	
	colistin			At 15 × MIC, the PAS effect on biofilm degradation, and at 2 × MIC, the PAS effect on inhibition of biofilm formation was observed	

Abbreviations: PAS—phage–antibiotic synergy; P—podovirus; M—myovirus; S—siphovirus; nd—no data.

**Table 4:** Activity of phage-derived enzymes on *A. baumannii* biofilm.

Type of Enzyme(s)	Phage Which Is the Source of the Enzyme	Description of Observed Activity	Reference
polysaccharide depolymerase	tailspike protein of phage φAB6	A 48 h biofilm formed in vitro by the *A. baumannii* strain Ab-54149 was treated with three different concentrations of the tailspike protein (10, 50, and 100 ng/well) for 4 h. The biofilm removal was ~9%, ~35%, and ~38%, whereas the biofilm inhibition demonstrated significant results: ~34%, ~44%, and ~56%, respectively.	[55]
Abtn-4	phage vB_AbaP	The lowest biofilm inhibition (~35%) was observed within the *A. baumannii* strains ATCC 17978 and ATCC 19606. The highest results (almost 70% biofilm reduction) were observed for *A. baumanii* strains AB7, AB10, and AB16. The inhibiting activity of Abtn-4 was also evaluated on a biofilm formed by bacterial strains belonging to other species (*K. pneumoniae*, *P. aeruginosa*, *Salmonella* sp., *E. faecium*, *E. faecalis*, and *S. aureus*); however, within all tested pathogens, it did not reach 50%.	[198]
endolysin PlyF307	a prophage induced from *A. baumannii* strain 2198	The enzyme activity was tested on biofilm formed by the *A. baumannii* strain 1791 in vitro (on catheter) and in vivo (in mice; infected by a small incision and later an insertion of a 3 cm catheter containing a 2-day preformed biofilm). After 2 h of the treatment, the bacterial density was decreased by 1.6 log units (in vitro; 1 dose—300 μL of 1 mg/mL solution of endolysin PlyF307), and after 3 h of the treatment by 2 log units (in vivo; 2 doses—250 μL of 4 mg/mL solution of endolysin PlyF307). The anti-biofilm effect of proteins P307 and P307_SQ-8C_ (P307 with additional disulfide bond, C-terminal proteins derived from PlyF307 endolysin) was assessed. After 2 h, a 3-log-unit, and a 4-log unit decrease in bacterial density was noted for P307 andP307_SQ-8C_, respectively.	[209]
LysAB3	phage AB3, and LysAB3 was subjected to a knock-out of a structural amphiphilic peptide region (forming LysAB3-D)	The decrease in antibacterial activity (between intact LysAB3 and LysAB3-D) was statistically relevant—from 95.8% to 33.3% of inhibiting activity.	[210]
modified lysin LysAB2-KWK	nd	LysAB2-KWK, with additional CeA peptide octamer, potentially enhanced lytic activity against an MDR-AB2 strain. The use of this enzyme reduced biofilm by ~40%. There was no difference after increasing the concentration of this enzyme.	[211]
endolysins: LysAm24, LysAp22, LysECD7, and LysSi3	myovirus	The anti-biofilm activity of endolysins was assessed on mature biofilms formed by the *A. baumannii* strain Ts 50–16. Inhibition of biofilms formed by different pathogens (*K. pneumoniae* and *P. aeruginosa*) was observed; the biofilm formed by *A. baumannii* was the most susceptible to these endolysins. The highest anti-biofilm activity was observed for the LysECD7 at 1 mg/mL concentration (decrease in OD_600_ from 1 to ~0.15). LysAm24 and LysAp22 also demonstrated great biofilm reduction (decrease in OD_600_ from 1 to ~0.2). The lowest anti-biofilm activity was observed fot LysSi3 (decrease in OD_600_ from 1 to ~0.5), suggesting that it was the least optimal enzyme in the eradication of biofilm.	[201]
lysin Abp013	phage φAbp2	A 3 h biofilm formed by the *A. baumannii* strain ATCC 17961 was reduced by 2.65 log units (99.78%), 2.23 log units (99.42%), and 1.51 log units (96.93%)—for the Abp013 concentration of 400, 800, and 1600 µg/mL, respectively. Although the mature (24 h) biofilm formation was more resistant to Abp013, the endolysin was able to reduce the CFU by 0.827 log units (85.13%) and 0.777 log units (83.32%)—for the Abp013 concentrations of 800 and 1600 µg/mL, respectively.	[212]
virion-associated lysins (VAL), PG_binding_3 domain-containing protein, putative endolysin, putative chitinase, carboxypeptidase, 1,4-beta-*N*-acetylmuramidase, putative lytic murein transglycosylase, and lysozyme	nd	Of the VAL proteins, only 1 of 3 was able to inhibit biofilm formation (with antimicrobial peptide MTKIGKRFRTKN). Furthermore, putative endolysin, putative chitinase, carboxypeptidase, and putative lytic murein transglycosylase were all 1 of 2 in terms of acting as an inhibitor of *A. baumannii* biofilms—GFRRKRPVSKYNKQQYIA (putative endolysin), GFRRKRPVSKYNKQQYIA (putative chitinase), FRRKRPVSKYNKQQYIA (carboxypeptidase), GWKHQRGALYSRNVLKKANY (putative lytic murein transglycosylase). All three members (PG_binding_3 domain-containing protein (LVRVLNIMQGQR), 1,4-beta-*N*-acetylmuramidase (KGRKSKVINSKGL), and lysozyme (LKYKYVAKRDCS)) had anti-biofilm activity. Importantly, all these lytic peptides were defined as not toxic to other cells (e.g., lacking poisonous placements of amino acids like His, Cys, Pro, Asn).	[213]
lysAB-vT2 fused with the hydrophobic amino acid at the C-terminus	phage vB_AbaM_PhT2	The highest density decrease (by 1.5 log unit) of mature biofilm formed by *A. baumannii* strain AB183 by lysAB-vT2-fusion was observed at 4 µg/mL concentration. It was similar to the activity of the vPhT2 (Φ2) phage (from which the endolysin was derived) with the titer 10^8^ PFU/mL. 2 µg/mL, and lower concentrations of the lysAB-vT2-fusion showed increased biofilm formation by the *A. baumannii* strain AB183 compared to the control sample.	[214]
endolysin Kp84B, linked to ApoE23 and COG133 peptides	phage ФKp84B	Chimeric protein significantly decreased the biofilm bacterial OD_600_ from 0.7 to 0.2. Moreover, it reduced the log unit of persister cells from 6.0 to 4.5 compared to the non-treated control sample.	[215]
depolymerase Dpo1	phage Petty (podovirus)	Dpo1 was able to disrupt biofilms formed by many tested *A. baumannii* strains; the results were not satisfying, since this protein degraded biofilm maximally by ~20%. Interestingly, the biofilm formed by the host of a Petty phage (AU0783) was the most resistant to the activity of Dpo1.	[216]

Abbreviation: nd—no data.

## Data Availability

Not applicable.
